# The Incidence, Severity and Clinical Consequence of Left Pulmonary Artery Stenosis After PDA Device Closure in Premature Infants Less Than 2 Kilograms

**DOI:** 10.1002/ccd.70849

**Published:** 2026-09-13

**Authors:** Bryan Mosher, Robin Wolschendorf, Preston Boyer, Jimmy C. Lu, Ray Lowery, Sunkyung Yu, Ashley Duimstra, Jeffrey D. Zampi

**Affiliations:** ^1^ Congenital Heart Center, C.S. Mott Children's Hospital University of Michigan Ann Arbor Michigan USA; ^2^ Stanford Cardiovascular Institute Stanford University School of Medicine Stanford California USA

## Abstract

**Background:**

Transcatheter patent ductus arteriosus (PDA) device closure is an effective treatment option for premature infants with hemodynamically significant PDA. Left pulmonary artery (LPA) stenosis has been reported as a complication, however there is limited data on risk factors, clinical significance, and long‐term outcomes. We aim to determine the incidence of LPA stenosis after transcatheter PDA closure in premature infants, describe anatomic factors associated with LPA stenosis, and evaluate the impact of LPA stenosis on pulmonary artery growth and re‐interventions over time.

**Methods:**

Single center retrospective cohort study of 116 premature infants who underwent transcatheter PDA closure while less than 2 kg. Clinical, angiographic and serial echocardiographic data were collected to determine the presence, severity, and clinical impact of LPA stenosis. Patient factors associated with the occurrence of LPA stenosis were evaluated using standard descriptive statistics.

**Results:**

LPA stenosis occurred in 43 of 116 patients (37%) within 1 day post‐procedure, though none had severe stenosis precluding device placement. At the most recent follow‐up (median 7.9 months), the degree of LPA stenosis improved in nearly all patients. Only one patient developed late, severe LPA stenosis related to device embolization post‐procedure. LPA stenosis was not associated with patient or procedural factors, and the presence of LPA stenosis was not associated with worse LPA growth.

**Conclusion:**

LPA stenosis is common after PDA device closure in premature infants but was infrequently associated with persistent obstruction or the need for intervention during the available follow‐up period. Most cases resolved over time without adverse impact, suggesting that its presence should not preclude device closure in this high‐risk group.

## 1 Introduction

A persistent patent ductus arteriosus (PDA) is very common in premature neonates, occurring in 33% of infants born less than 1500 g. [1] As PDAs are associated with heart failure, chronic lung disease, necrotizing enterocolitis, intraventricular hemorrhage, and death in premature infants, nearly 50% of those born less than 1000 g will require PDA closure. [1, 2, 3] While medical therapy remains the first‐line treatment to induce ductal constriction and closure, if ineffective or contraindicated, surgical ligation or transcatheter device closure is often performed. [4, 5] Transcatheter device closure has become increasingly common, as recent studies found transcatheter PDA closure to be as effective as surgical ligation while offering less morbidity [4, 6, 7].

While transcatheter PDA device closure offers several advantages over surgical ligation for premature infants, it also carries unique risks. Left pulmonary artery (LPA) stenosis can occur after PDA device closure and can range from trivial compression to severe obstruction. There is limited data to identify risk factors for LPA stenosis, and little knowledge about the clinical impact of LPA stenosis in these premature infants [8, 9]. Although prior studies have described the occurrence of LPA stenosis after transcatheter PDA closure [10, 11], data regarding risk factors, long‐term pulmonary artery (PA) growth, and the natural history of stenosis in contemporary cohorts of extremely premature infants remain limited.

We aimed to describe the incidence and severity of LPA stenosis after PDA device closure in a cohort of premature infants less than 2 kg, examine anatomic factors associated with LPA stenosis, and describe the clinical implications of LPA stenosis, including the impact on branch PA growth and need for PA interventions.

## 2 Methods

This single‐center retrospective cohort study included all premature infants who underwent attempted PDA device closure at less than 2 kg between 2016 and 2023. Patients were excluded if they had congenital heart defects other than a PDA, including preexisting LPA stenosis or aortic obstruction. Patient demographic information was collected from chart review, including gestational and current age, birth and current weight, prior medical treatment for attempted PDA closure, and current respiratory support. Procedural details were collected from cardiac catheterization reports, including classification of the PDA morphology and device used for closure, as well as any procedural or post‐procedural complications. The pre‐procedural, intraprocedural, and follow‐up echocardiograms and angiographic imaging were reviewed by members of the study team. From the lateral angiographic images (all taken at LAO‐90, Cranial‐0 projection), the angle between the PDA and the LPA was measured by creating a straight line to simulate the major axis of both the PDA and the LPA (Figure 1). This was done using a free online protractor tool software [12]. Post‐procedure Day 1 echocardiograms (for all patients) were reviewed to assess the presence and severity of LPA stenosis, as well as measure the LPA and right PA (RPA). In addition, the most recent echocardiograms, when available, were reviewed to assess long‐term growth. The parasternal short axis was used as a standard for measuring the LPA and RPA diameters. *Z*‐scores were obtained using the Pediatric Heart Network model [13].

**Figure 1:**
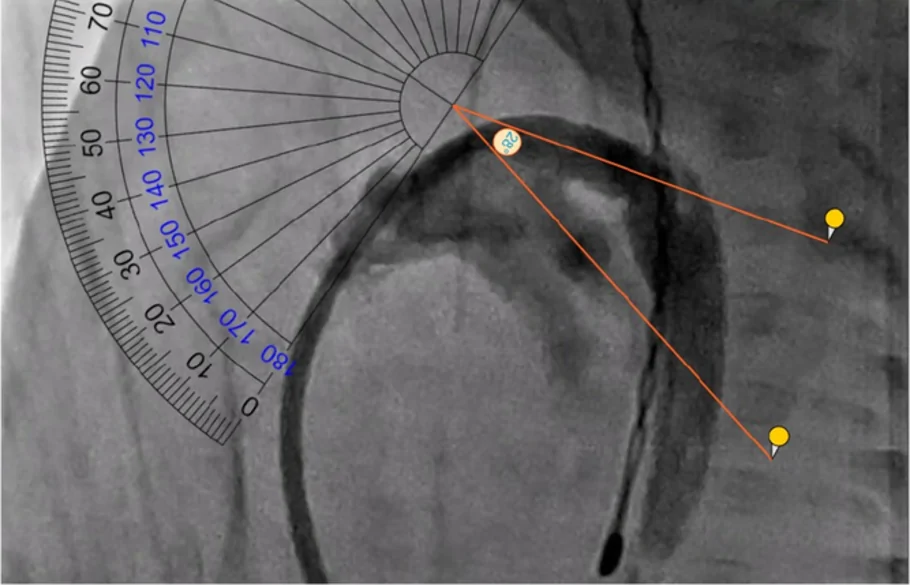
Angle measurement in baseline PDA angiogram. Using an online protractor tool [12], the angle between the central axis of the LPA and the portion of the ductus in which the device was subsequently deployed was measured. [Color figure can be viewed at wileyonlinelibrary.com]

All echocardiograms were reviewed by two independent pediatric cardiologists for the purposes of determining inter‐observer reliability in LPA measurements after PDA device placement. Reviewers independently assessed branch PA dimensions while blinded to the assessments of the other reviewer. Interobserver agreement was assessed using weighted kappa statistics for categorical variables and intraclass correlation coefficients with 95% confidence intervals for continuous variables. For continuous measurements, the mean of the two reviewers' measurements was used for subsequent analyses. The degree of LPA stenosis was determined by independent review of the echocardiographic images and classified as none, trivial, mild, moderate, or severe based on an integrated assessment of two‐dimensional imaging and color Doppler findings.

To further analyze LPA stenosis on the immediate post‐PDA closure echocardiogram, patients were grouped into none or trivial LPA stenosis and mild or worse LPA stenosis. No prespecified Doppler velocity or diameter thresholds were used to define severity categories.

### 2.1 Statistical Analysis

Standard descriptive statistics (mean with standard deviation or median with interquartile range) were reported for continuous variables, and counts with percentages for categorical variables were reported. Outcomes were presented as rates along with 95% confidence intervals. As a secondary analysis, the associations between various patient characteristics (demographic, clinical, anatomic, and procedural factors) and primary outcomes were evaluated. Differences between subjects with each outcome were compared using Student's *t*‐test, Wilcoxon rank, or chi‐square tests as appropriate. The threshold for statistical significance was set at *p* < 0.05. For interrater reliability, the weighted kappa was reported for categorical variables and intraclass correlation with 95% confidence interval was used for continuous variables. To examine the influence of normal versus low normal or hypoplastic branch pulmonary arteries on the primary outcome of LPA stenosis after PDA closure, cut‐offs were made for both the LPA and RPA *z*‐scores based on the cohort data.

## 3 Results

The baseline characteristics of the 116 patients who had attempted PDA closure are described in Table 1. The study cohort, procedural success, and availability of follow‐up echocardiography are summarized in Figure 2. The average birth weight was 700 g, and gestational age was 24 weeks. The vast majority received medical treatment prior to attempted PDA device closure. The median age in months at PDA closure was 1.1 (range 0.90−1.6 months) with an average weight of 1.1 ± 0.34 kg. The majority of patients had type C PDA morphology. As shown in Table 2, most PDAs were closed using the Amplatzer Piccolo occluder versus with the Amplatzer Duct Occluder II. One patient had unsuccessful PDA closure, which was secondary to early device embolization requiring surgical closure. This patient was excluded from further analyses. Otherwise, procedural and post‐procedural complications were rare with an overall complication rate of 5.2%. One additional patient experienced late device malposition resulting in complete LPA occlusion requiring surgical intervention (see discussion below).

**Table 1:** Demographics and pre‐procedural characteristics (*N* = 116).

Male sex	43 (37.1)
Caucasian race	78 (67.2)
Hispanic ethnicity	5 (4.3)
Birth weight, kg	0.70 ± 0.18
Gestational age at birth, weeks	24 (23−26)
*Respiratory support immediately prior to PDA closure*	
RA	0 (0.0)
HFNC	1 (0.9)
CPAP	30 (25.9)
Mechanical vent	74 (63.8)
Oscillator	1 (0.9)
JET	7 (6.0)
NC	2 (1.7)
NIV/NAVA	1 (0.9)
Tracheostomy pre‐PDA closure	1 (0.9)
Inhaled nitric oxide pre‐PDA closure	6 (5.2)
Medical treatment pre‐PDA closure	100 (86.2)
*Number of medical treatment cycles pre‐PDA closure*	
1	46/100 (46.0)
2	41/100 (41.0)
3	13/100 (13.0)
Ibuprofen pre‐PDA closure	67/100 (67.0)
Total duration of ibuprofen pre‐PDA closure, days (*N* = 66)	4 (3−5)
Acetaminophen pre‐PDA closure	52/100 (52.0)
Total duration of acetaminophen pre‐PDA closure, days (*N* = 52)	7 (4.5−8.5)
Age at PDA closure, months	1.1 (0.90−1.6)
Weight at PDA closure, kg	1.1 ± 0.34
Height at PDA closure, cm	35.3 ± 3.6
BSA at PDA closure, m^2^	0.11 ± 0.02
Pre‐closure PDA morphology	116 (100.0)
A	11 (9.5)
B	0 (0.0)
C	66 (56.9)
D	2 (1.7)
E	30 (25.9)
F	41 (35.3)
Pre‐closure PDA/LPA angle, degrees	16 (8−25)

*Note:* Data are presented as *N* (%) for categorical variables and mean ± standard deviation or median (interquartile range) for continuous variables.

**Figure 2:**
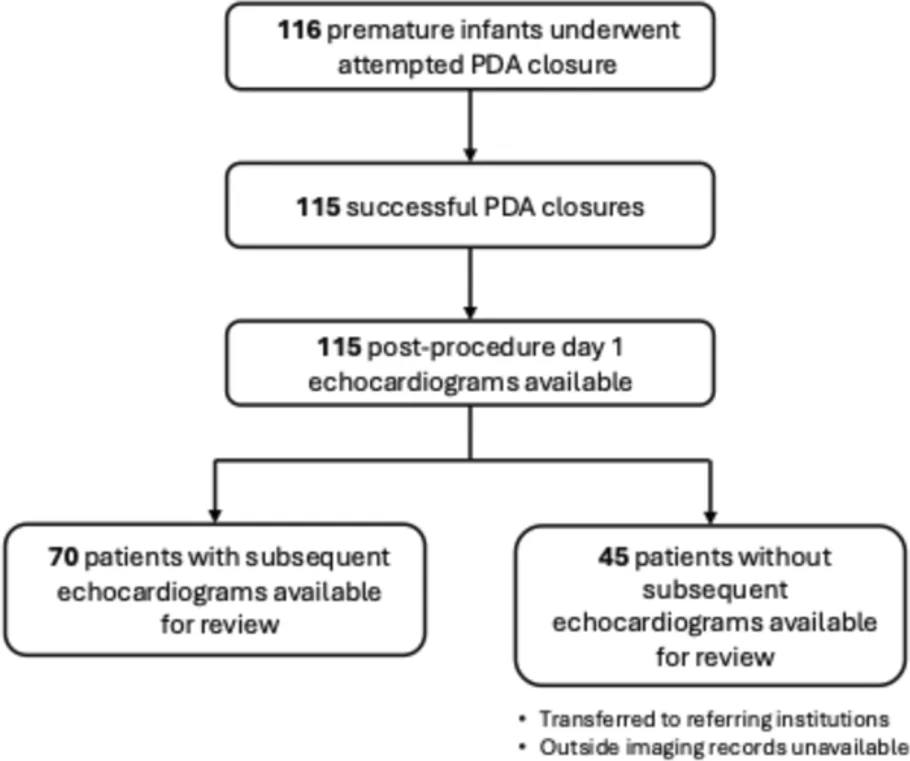
Study cohort and availability of follow‐up echocardiography. Among 116 premature infants who underwent attempted transcatheter PDA closure, one procedure was unsuccessful due to early device embolization requiring surgical ligation. Post‐procedure Day 1 echocardiograms were available for all patients with successful transcatheter PDA closure. Subsequent echocardiograms were available for review in 70 patients (61%), while 45 patients (39%) did not have follow‐up imaging available, primarily because they had been transferred back to referring institutions and outside imaging records could not be obtained.

**Table 2:** Procedural characteristics (*N* = 116).

*Closure device type*	
Piccolo	104 (89.7)
Amplatzer vascular plug	12 (10.3)
*Piccolo size (N = 104)*	
3−2	36 (34.6)
3−4	1 (1.0)
3−6	0 (0.0)
4−2	56 (53.8)
4−4	3 (2.9)
4−6	0 (0.0)
5−2	6 (5.8)
5−4	2 (1.9)
5−6	0 (0.0)
*Amplatzer vascular plug size (N = 12)*	
4	4 (33.3)
6	8 (66.7)
8	0 (0.0)
Unsuccessful PDA closure	1 (0.9)
Post‐closure complication	6 (5.2)
Device malposition or embolization	2 (1.7)
Bleeding requiring transfusion	1 (0.9)
Arrhythmia	0 (0.0)
Increase to moderate or severe tricuspid regurgitation (not back to baseline pre‐discharge)	3 (2.6)
Vascular—arterial complication	1 (0.9)
Vascular—venous complication	0 (0.0)

*Note:* Data are presented as *N* (%) for categorical variables and mean ± standard deviation or median (interquartile range) for continuous variables.

During device closure, intraprocedural echocardiogram was concerning for LPA compression in 38 patients (33.0%) (Figure 3A, Table 3). However, none were severe enough to warrant device removal or unsuccessful PDA closure. The time course of degree of LPA stenosis is demonstrated in Figure 3. On the first post‐procedure day echocardiogram, 72 patients had no or trivial LPA stenosis (62.6%), 40 had mild stenosis (34.8%), three patients had moderate stenosis (2.6%), and no patients had severe stenosis (Figure 3A, Table 3). A subset of patients was transported back to their previous institutions, where subsequent imaging records were unavailable. Consequently, 70 patients had subsequent echocardiograms available for review at a median of 7.9 months (interquartile range 4.1−19.7 months) following PDA closure, while 45 patients did not have subsequent echocardiograms available for review, primarily because they had been transferred back to their referring institutions and outside imaging records could not be obtained (Figure 2). Of the 70 patients, 52 (74.2%) had no LPA stenosis, eight (11.4%) had trivial stenosis, eight (11.4%) had mild stenosis, zero had moderate stenosis, one patient had severe LPA stenosis (1.4%), and one patient (1.4%) had an unknown degree of LPA stenosis on most recent echocardiogram (Figure 3A, Table 3) because the patient was transferred back to previous hospital and records could not be obtained. Of the 70 patients with subsequent echocardiograms, 21 patients had mild LPA stenosis on the first post‐closure echocardiogram, and one patient had moderate LPA stenosis (Figure 3B). These two groups of patients representing mild/moderate LPA stenosis at first post‐closure echocardiogram accounted for 31% of patients who also had subsequent echocardiograms available for review (Figure 3B). This same group of patients had an improvement of LPA stenosis at subsequent echocardiograms, with mild or worse LPA stenosis in only 13% of patients at most recent evaluation (Figure 3B).

**Figure 3:**
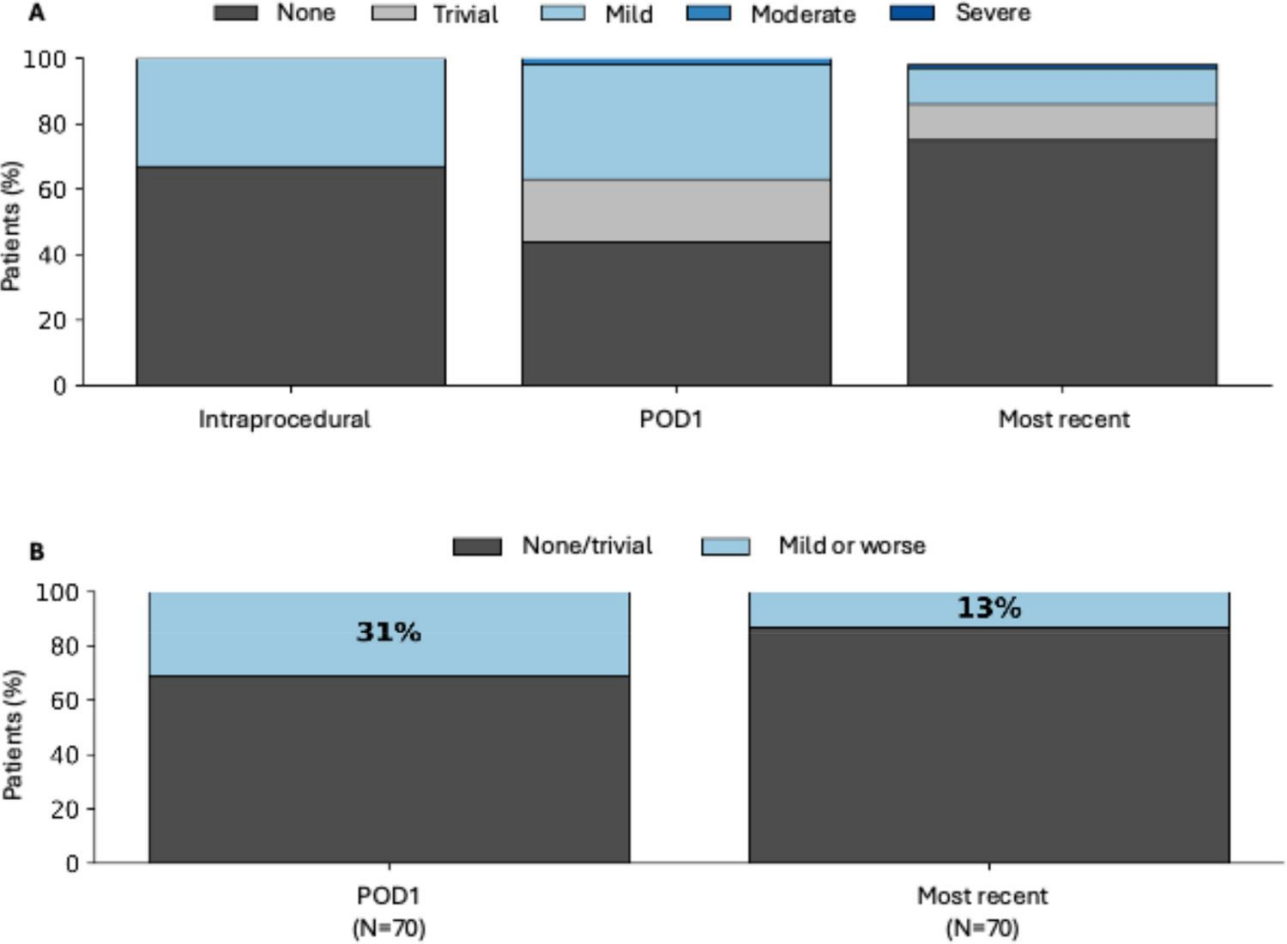
Evolution of left pulmonary artery (LPA) stenosis following transcatheter PDA closure. (A) Distribution of LPA stenosis severity at the time of intraprocedural echocardiography, on the first post‐procedure day (POD1) echocardiogram, and at the most recent follow‐up echocardiogram in the entire study cohort (*N* = 115). (B) Distribution of none/trivial versus mild or worse LPA stenosis among patients with echocardiograms available for review on POD1 (*N* = 70) and most recent (*N* = 70). The proportion of patients with mild or worse LPA stenosis decreased from 31% on the POD1 echocardiogram to 13% at the most recent evaluation. [Color figure can be viewed at wileyonlinelibrary.com]

**Table 3:** Echocardiographic assessment of branch pulmonary arteries.

Concern for LPA compression or stenosis during procedure 38 (33.0)	
**First post‐PDA closure echo (*N* = 115)**	
Time from PDA closure to echo, days	1 (1−1)
Weight at echo, kg	1.2 ± 0.41
Height at echo, cm	35.4 ± 3.7
BSA at echo, m^2^	0.11 ± 0.02
LPA diameter, mm	3.25 ± 0.62
LPA diameter *z*‐score (PHN)	−1.25 ± 0.85
RPA diameter, mm	3.6 ± 0.62
RPA diameter *z*‐score (PHN)	0.16 ± 0.89
*LPA stenosis*	
None	50 (43.5)
Trivial/compression	22 (19.1)
Mild	40 (34.8)
Moderate	3 (2.6)
Severe	0 (0.0)
**Most recent echo (N = 70)**	
Time from PDA closure to echo, months	7.9 (4.1−19.7)
Weight at echo, kg	7.3 ± 3.8
Height at echo, cm	65.3 ± 16.5
BSA at echo, m^2^	0.36 ± 0.14
LPA diameter, mm	5.85 ± 1.8
LPA diameter *z*‐score (PHN) (*N* = 70)	‐0.95 ± 0.98
RPA diameter, mm	6.6 ± 1.75
RPA diameter *z*‐score (PHN) (*N* = 70)	0.29 ± 0.98
*LPA stenosis*	
None	52 (74.2)
Trivial/compression	8 (11.4)
Mild	8 (11.4)
Moderate	0 (0.0)
Severe	1 (1.4)
Unknown	1 (1.4)

*Note:* Data are presented as *N* (%) for categorical variables and mean ± standard deviation or median (interquartile range) for continuous variables. LPA/RPA measurements represent the mean of reviewers.

Categorization of degree of LPA stenosis had excellent interrater reliability, with Kappa 0.96 on the first post‐PDA echocardiogram and 1.0 on the most recent post‐PDA echocardiogram.

Comparing those patients with none/trivial LPA stenosis (*N* = 72) to those with mild/moderate LPA stenosis (*N* = 43) on the first post‐procedure Day 1 echocardiogram, there were no significant differences in demographic, clinical, procedural, or angiographic characteristics, including no differences in PDA morphology, pre‐closure PDA/LPA angle on angiography, or device used for closure.

On serial echocardiograms, the vast majority of patients had improvement in the degree of stenosis (Figure 3, Table 3). Only 18 patients (16%) had an increase in stenosis, and all were from none/trivial in the intraprocedural echocardiogram to mild at the 1st post‐procedure day echocardiogram. Of these 18, eight improved back to none/trivial on follow‐up echocardiogram, two remained mild, and one became severe (patient with late device malposition as described above).

Looking at branch PA sizes and PA growth, both LPA and RPA *z*‐scores were significantly lower in those patients with mild or moderate LPA stenosis (Table 4). For those with recent echocardiograms (*N* = 70), mild or moderate LPA stenosis was only associated with significantly lower LPA *z*‐scores, but no difference in RPA *z*‐scores compared to those with no or trivial LPA stenosis. PA *z*‐scores remained stable in all patients, indicating PA growth; patients with more LPA stenosis had a far greater increase in RPA *z*‐scores (Table 4).

**Table 4:** (Univariate) associations of PA diameter *z*‐scores (PHN) with LPA stenosis.

	None or trivial/compression LPA stenosis	Mild, moderate, or severe LPA stenosis	*p* value^a^
First post‐PDA closure echo (*N* = 115)			
LPA diameter *z*‐score (PHN)	−1.0 ± 0.88	−1.5 ± 0.79	0.001
RPA diameter *z*‐score (PHN)	0.31 ± 0.87	−0.10 ± 0.94	0.02
Most recent echo (*N* = 70)			
LPA diameter *z*‐score (PHN)	−0.81 ± 0.95	−1.6 ± 0.91	0.02
RPA diameter *z*‐score (PHN)	0.29 ± 0.83	0.30 ± 2.2	0.98

*Note:* Data are presented as mean ± standard deviation;

^a^ *p* value from two‐sample *t*‐test for continuous variables.

Lastly, based on the branch PA *z*‐score data from our cohort, patients with low‐normal branch PA sizes (LPA *z*‐score < −0.6 and RPA *z*‐score < 0 [*N* = 51]) were more likely to have mild or worse LPA stenosis on the post‐procedure Day 1 and late echocardiograms compared to those with more normal branch PA sizes (LPA *z*‐score ≥ −0.6 and RPA *z*‐score ≥ 0 [*N* = 64]) (Table 5).

**Table 5:** (Univariate) associations of LPA stenosis with PA diameter *z*‐scores (PHN).

	LPA *z*‐score < −0.6 and RPA *z*‐score < 0 (*N* = 51)	LPA *z*‐score ≥ −0.6 and/or RPA *z*‐score ≥ 0 (*N* = 64)	*p* value^a^
LPA stenosis at first post‐PDA closure echo			0.01^b^
None	17 (33.3)	33 (51.6)	
Trivial/compression	8 (15.7)	14 (21.9)	
Mild	23 (45.1)	17 (26.6)	
Moderate	3 (5.9)	0 (0.0)	
Severe	0 (0.0)	0 (0.0)	

		LPA *z*‐score < −0.6 and RPA *z*‐score < 0 (*N* = 18)	LPA *z*‐score ≥ –0.6 and/or RPA *z*‐score ≥ 0 (*N* = 52)	*p* value^a^
LPA stenosis at most recent echo				0.04^b^
None		10 (55.6)	43 (82.7)	
Trivial/compression		3 (16.7)	5 (9.6)	
Mild		5 (27.8)	3 (5.8)	
Moderate		0 (0.0)	0 (0.0)	
Severe		0 (0.0)	1 (1.9)	

*Note:* Data are presented as *N* (%).

^a^ *p* value from Chi‐square test or Fisher's exact test for categorical variables.

^b^ Comparison made for Chi‐square test or Fisher's exact test was none and trivial/compression versus mild, moderate, and severe.

Other than the aforementioned patient who developed late severe LPA stenosis, no patient required any interventions related to LPA stenosis.

## 4 Discussion

As transcatheter closure of PDAs in premature infants becomes a common alternative to surgical ligation and a second‐line treatment for those who have failed or are contraindicated to undergo medical therapy, we must better understand the risks and benefits of this treatment option. Consistent with prior literature, we report that PDA device closure in those less than 2 kg is very effective with low risk of major complications. However, LPA stenosis is an important risk of this procedure, and our findings provide valuable insight into the occurrence and clinical significance of LPA stenosis.

Our findings are consistent with prior studies from both the pre‐Piccolo and Piccolo eras demonstrating that flow acceleration or mild LPA stenosis is common after transcatheter PDA closure but frequently improves over time without intervention. Previous investigations have reported a low incidence of clinically significant branch PA obstruction and have similarly shown that most cases resolve or remain stable during follow‐up [10, 11]. The present study expands upon these observations by providing detailed longitudinal echocardiographic assessment of branch PA growth and by evaluating demographic, anatomic, and procedural factors associated with the development of LPA stenosis in a contemporary cohort of premature infants weighing less than 2 kg.

Consistent with prior reports, we found that the total incidence of LPA stenosis after PDA closure in premature infants less than 2 kg is as high as 37%. Fortunately, the clinical implications of LPA stenosis appear very limited. When present, stenosis was at worst moderate, and the vast majority of infants exhibited improvement in the degree of LPA stenosis over time. This could be related to changes in the relative position of the PDA device to the branch PAs over time. Most LPA stenosis is related to compression of the LPA in the superior‐inferior dimension. With somatic growth, these two structures likely grow further away, mitigating some of this compression. Further, branch PA growth appears largely preserved in these patients regardless of the degree of LPA stenosis, and PDA device size remains fixed. There was no significant difference in RPA *z*‐scores between those with and without LPA stenosis, indicating that RPA growth is less affected. Therefore, while LPA stenosis is important to recognize and document at the time of PDA device closure, our experience suggests that mild to moderate degrees of stenosis frequently improve over time and can often be managed with careful surveillance.

Importantly, serial assessment of branch PA sizes and flow is crucial in this high‐risk patient population. As noted above, although PA *z*‐scores remained stable in all patients, indicating PA growth, patients with more LPA stenosis had a far greater increase in RPA *z*‐scores. This could indicate clinically significant differences in branch PA flow leading to discrepant PA growth. Therefore, we recommend that all infants who undergo PDA closure as premature infants undergo echocardiographic evaluation throughout childhood to ensure appropriate PA growth. This demonstrates the need for further studies to understand how even mild stenosis may be related to PA growth.

Serial assessment of branch PA growth and flow is important because late device malposition or embolization can occur, potentially resulting in severe LPA stenosis, as was seen in one of our patients [7, 14]. In this case, although being followed in a neonatal intensive care unit, flow in the LPA was difficult to assess by echocardiogram, which eventually prompted a CT, which showed complete LPA occlusion. This may represent underappreciation for severe narrowing, in the context of difficult echocardiographic windows, rather than true progression. Although surgical removal of the device was successful, there was significantly diminished size/growth of the LPA requiring surgical shunt placement and serial cardiac catheterization procedures for rehabilitation. Therefore, we recommend serial echocardiography with a low threshold to obtain axial imaging to evaluate differential pulmonary flow or nuclear perfusion scans if there is any concern for decreased flow to a branch PA.

We did not identify any clinical characteristics that were significantly associated with higher grade LPA stenosis at the time of implant or at subsequent evaluations, although the study may have been underpowered to detect modest associations. We had postulated that understanding the PDA morphology and its relationship to the branch PAs would have been a useful tool to predict LPA stenosis. While this was not the case here, we will continue to evaluate ways to anticipate potential complications during high‐risk procedures, like PDA closure in premature infants. As one might have expected, patients with smaller branch PAs at baseline were more likely to have LPA stenosis after PDA device closure (in the example above, 1 mm compression in a 2.5 mm vessel is 40% luminal loss). So, this may be one group in particular who should be monitored more closely for LPA stenosis during and after PDA closure.

## 5 Limitations

Intraprocedural echocardiograms present unique challenges, including the sonographer's hand needing to be under surgical drapes, limited positioning, and difficult probe access. As such, comparing intraprocedural echocardiograms to postoperative echocardiograms may be difficult, and although standard techniques and methods of measuring and describing branch PA sizes and degrees of stenoses were used, there can be some subjectivity, especially in patients less than 2 kg. We have attempted to account for this using multiple independent reviewers with comparisons using interrater variability statistics, but this could impact the ability to replicate the exact findings across centers. Long‐term echocardiographic follow‐up was unavailable in approximately 39% of the cohort, primarily because many infants were transferred back to referring institutions. Consequently, the true incidence of persistent or late LPA stenosis may be under‐ or overestimated.

Although this represents one of the larger single‐center cohorts of premature infants undergoing transcatheter PDA closure, the modest sample size and low number of clinically significant events limited our statistical power to identify factors associated with persistent or severe LPA stenosis. Consequently, negative findings should be interpreted cautiously, as type II error is possible and residual confounding cannot be excluded. We have demonstrated that LPA stenosis improves over time, likely related to PA growth; however, future studies are needed to follow these patients throughout childhood to specifically assess PA growth, re‐intervention burden, or other unexpected consequences related to PDA closure in infancy. Because decisions regarding additional imaging and intervention were made at the discretion of the treating clinicians, the absence of intervention should not be interpreted as proof that mild residual stenosis lacked clinical significance.

## 6 Conclusion

PDA device closure in premature infants weighing less than 2 kg is very feasible with low rates of procedural complications. LPA stenosis is frequently seen during or immediately after device closure but is of limited clinical significance in the vast majority of patients. However, continued screening is important to document any branch PA stenosis and assure acceptable branch PA flow and growth.

## Conflicts of Interest

The authors declare no conflicts of interest.

## Data Availability

The data that support the findings of this study are available from the corresponding author upon reasonable request.
